# Is microRNA 1910-3p (miR-1910-3p) a really distinctive marker for psoriasis?

**DOI:** 10.3906/sag-2009-156

**Published:** 2021-06-28

**Authors:** Melek KARABACAK, İjlal ERTURAN, Kuyaş HEKİMLER ÖZTÜRK, Havva Hilal AYVAZ, Selma KORKMAZ, Mehmet YILDIRIM, Hikmet ORHAN

**Affiliations:** 1 Department of Dermatology, Faculty of Medicine, Süleyman Demirel University, Isparta Turkey; 2 Department of Medical Genetics, Faculty of Medicine, Süleyman Demirel University, Isparta Turkey; 3 Department of Biostatistics and Medical Informatics, Faculty of Medicine, Süleyman Demirel University, Isparta Turkey

**Keywords:** Psoriasis, miR-1910-3p, IL-17A, regulatory T-cells

## Abstract

**Background/aim:**

Although the cause of immune activation in the pathogenesis of psoriasis is still unclear, miRs are thought to have an effect on psoriasis. This work aimed to evaluate the role of miRs (miR-4649-3p, miR-6867-5p, miR-4296, miR-210, and miR-1910-3p) that target the FOXP3 mRNA and IL-17A mRNA in psoriasis.

**Materials and methods:**

Forty-four psoriasis patients and 44 healthy controls were included in the study. Quantitative real-time PCR (qRT-PCR) was used for the measurement of miRs. Serum IL-17A levels were determined by an enzyme-linked immunosorbent assay (ELISA) method.

**Results:**

Plasma miR-1910-3p levels were significantly lower in the patient group than the controls (P = 0.000, fc: 0.10). ROC analysis showed that plasma miR-1910-3p levels could significantly differentiate psoriasis patients from healthy controls [AUC = 0.912 (0.848–0.975), P = 0.000]. The plasma miR-4649-3p level was significantly higher in the psoriasis group compared to the controls (P = 0.000, fc: 2.99).

**Conclusion:**

Decreased expression of miR-1910-3p increases the risk of developing psoriasis by approximately 50-fold and was able to use for the significant differentiation of psoriatic patients from healthy controls.

## 1. Introduction

Psoriasis is a chronic, systemic inflammatory disease that affects approximately 1%–3% of the population world-wide. Cytokines, such as IL-17, IL-22, IL-12, IL-23, and tumor necrosis factor alpha (TNF-α) are secreted from T-helper lymphocytes (Th), especially Th1 and Th17 subtypes, which have many roles in the pathogenesis of psoriasis [1,2].

Regulatory T (Treg) cells suppress the immune activation via inhibition of Th1 and Th17 cells under homeostatic conditions. Disruption of the regulatory functions of Treg cells may lead to an increase the expression of IL-17A in psoriasis [3]. Although the underlying mechanism of Treg cell dysfunction has not yet been fully clarified yet, microRNAs (miRs) may be responsible for this situation [4]. miRs, a member of the noncoding RNA group, which have important regulatory roles in cell type identification and cell differentiation. Several studies have implicated miRs in the pathogenesis of psoriasis in recent years [4,5].

In the current study, it was aimed to evaulate the plasma levels of miRs, such as miR-4649-3p, miR-6867-5p, miR-4296, and miR-210 targeting the Treg cells in the patients with psoriasis and healthy controls. Additionally, the circulating levels of miR-1910-3p targeting the IL-17A mRNA as well as serum levels of IL-17A protein were measured. To the best of our knowledge, there has been no study examining the plasma levels of miR-4649-3p, miR-6867-5p, miR-4296, and miR-1910-3p in the patients with psoriasis so far.

## 2. Materials and methods

The study was initiated with the desicion of Süleyman Demirel University (SDU) Faculty of Medicine Clinical Research Ethics Committee (Date 04.07.2018, No:118). Forty-four psoriatic patients and 44 healthy controls over the age of 18 years, who were diagnosed with psoriasis clinically and/or histopathologically, were included in the study. Inclusion criteria included patients who had not received any systemic treatment for psoriasis for the last 3 months, had not received topical treatment for the last 3 weeks and had no inflammatory disease other than psoriasis. Healthy subjects without any known disease and not using any systemic drugs were included in the control group. Breastfeeding and pregnant women were excluded from the study.

Systematic data including age, sex, age at disease onset (early onset: ≤40 years, late onset: > 40 years), disease duration, family history, presence of arthritis, nail changes, smoking, and alcohol consumption were obtained from participants. The severity of the disease was evaluated by the Psoriasis Area Severity Index (PASI). The disease was classified as mild with a PASI score of ≤10 and as moderate-severe with PASI > 10.

### 2.1. Measurement of serum IL-17A levels

Peripheral venous blood samples were collected from the study group into appropriate tubes individually and centrifuged at 2500 rpm for 10 min. The supernatant was transferred to an Eppendorf tube and stored at –80 °C until use. The serum IL-17A levels were analyzed using an IL-17A human enzyme-linked immunosorbent assay (ELISA) kit (Elabscience, Wuhan, China) using a microplate reader (MRC, UT6100, Holon, Israel). 

### 2.2. miR expression and data analysis

Peripheral blood samples taken into EDTA tubes were centrifuged for 10 min at 2500 rpm within 2 h. The plasma was separated into Eppendorf tubes and stored at –80 °C until use. miRs from the plasma samples were isolated using a Hybrid-RTM miRNA isolation kit (GeneAll Biotechnology, Korea) according to the manufacturer’s instructions. Complementary DNA (cDNA) was synthesized from the isolated miRs using the HyperScriptTM reverse transcriptase kit (GeneAll Biotechnology, Korea). Reverse transcription was carried out using a SimpliAmp thermal cycler (Thermo Fisher Scientific Inc.,Waltham, MA, USA). Quantitative real-time polymerase chain reaction (PCR) reactions (qRT-PCR) were performed using a high-capacity StepOnePlus real-time PCR System (Thermo Fisher Scientific Inc.) according to the manufacturer’s instructions. All experiments were performed in triplicate. The expression levels of miRs were calculated using the 2-∆∆Ct method.

### 2.3. Primers

Amplification of the U6 gene was used as a housekeeping control reaction. The forward and reverse primers were designed on the basis of the criteria stated in quantitative stem loop RT-PCR for detection of miRs (Chapter 10) by Erika Varkonyi-Gasic and Roger P. Hellens. The primer sequences used in the study are shown in Table 1.

**Table 1:** Primary sequences used in the study.

miR	Primary squence
Hsa-miR-4649-3p	R: CGAGGAAGAAGACGGAAGAAT
	F: TCTGAGGCCTGCCTCTCCCCA
Hsa-miR-6867-5p	R: CGAGGAAGAAGACGGAAGAAT
	F: TGTGTGTGTAGAGGAAGAAGGGA
Hsa-miR-4296	R: CGAGGAAGAAGACGGAAGAAT
	F: ATGTGGGCTCAGGCTCA
Hsa-miR-210	R: CGAGGAAGAAGACGGAAGAAT
	F: AGCCCCTGCCCACCGCACACTG
Hsa-miR-1910-3p	R: CGAGGAAGAAGACGGAAGAAT
	F: GAGGCAGAAGCAGGATGACA
U6 snRNA	R: CGCTTCACGAATTTGCGTGTCAT
	F: GCTTCGGCAGCACATATACTAAAAT

## 3. Statistical analysis

SPSS statistical software (PASW Statistics for Windows, Version 20.0, SPSS Inc. IBM Corp., Chicago, IL, USA) was used to evaluate all statistical analyses. Continuous variables were expressed as mean ± SD and the level of significance was assigned as P < 0.05. Normality distributions of the samples was tested with the Kolmogorov–Smirnov test. ANOVA followed by post hoc tests were used to evaluate the group means. Independent sample t-test and Mann–Whitney U test were used for normally distributed variables and nonnormally distributed variables, respectively. Differences in the expression of miRNAs were calculated as fold changes using the 2-ΔΔCt equation. Each sample was run in triplicate for analysis. The expression levels of miRNAs were normalized to RNU6 (U6 small nuclear RNA). The ΔCt was calculated by subtracting the Ct values of RNU6 from the Ct values of the miRNA of interest. Receiver operating characteristic (ROC) curves were plotted to determine the strength of miRs in the classification of patient groups. Logistic regression analysis was used to determine the risk of developing the independent variables. ΔCT values of miRNAs were used for logistic regression analysis. Pearson correlation analysis was used to determine the association between between independent variables. Z-test was used to compare two independent proportions of categorical variables. 

Achieved power of the study was found to be 95% for results the area under the ROC curve (AUC) under the null hypothesis of 0.9120 using a two-sided significance level of 0.050. 

## 4. Results

The characteristics of the patient and control groups included in the study are given in Table 2. A comparison of the age at disease onset, PASI score, nail involvement, psoriatic arthritis, family history, and smoking habits of the patients with psoriasis is given in Table 3. The mean PASI score in male patients (11.01 ± 6.56) was significantly higher than the mean PASI score in female patients (6.23 ± 3.62) (P = 0.003). The ratio of mild (PASI < 10) psoriasis patients (63.6%) was significantly higher than the ratio of moderate-severe (PASI ≥ 10) patients (16, 36.4%) (P = 0.008). The number of patients with moderate-severe plaque psoriasis (PASI ≥10) was significantly higher in males than in females (P = 0.013). The mean PASI score (10.32 ± 6.60) of patients belonging to the early onset group was significantly higher than the mean PASI score of the patients who were classified as the late onset group (6.76 ± 3.76) (P = 0.030).

**Table 2:** Clinical and demographic characteristics, MiRNA ΔCT and IL-17A levels in patient and control groups.

	Patients (n: 44)	Controls (n: 44)	P	FC***
Male/female, n	28/16	28/16	1	-
Age, mean±SD	43.02 ± 16.26	42.82 ± 16,14	0.953*	-
Age onset of the disease, mean ± SD	31.36 ± 18.81	-	-	-
PASI, mean ± SD	9.27±6.08			
Female	6.23 ± 3.62	-		
Male	11.01 ± 6.56	-	0.003**	
Early onset	10.32 ± 6.60	-		
Late onset	6.76 ± 3.76	-	0.03**	
Duration of the disease, year, mean ± SD	11.65 ± 9.18	-	-	-
miR-1910-3p ΔCT, mean ± SD	14.54 ± 2.39	17.80 ± 0.87	0.000	0.10
miR-4649-3p ΔCT, mean ± SD	16.1 ± 1.97	14.63 ± 1.82	0.000	2.99
miR-4296 ΔCT, mean±SD	8.72 ± 2.25	9.17 ± 1.30	0.255	0.73
miR-210 ΔCT, mean ± SD	19.12 ± 1.69	18.94 ± 1.02	0.555	1.13
miR-6867-5p ΔCT mean ± SD	15.84 ± 1.80	15.54 ± 1.08	0.354	1.23
IL-17A, mean ± SD	167.03 ± 374.23	42.28 ± 25.51	0.033	-

**Table 3:** Clinical findings of psoriatic patients.

Clinical findings		n (%)	P*
Age at disease onset	Early onset (Type 1 < 40 year)	31 (70.5%)	0.000
	Late onset (Type 2 ≥ 40 year)	13 (29.5%)	
PASI	Mild (PASI <10)	28 (63.6%)	0.008
	Moderat-severe (PASI ≥10)	16 (36.4%)	
Nail involvement	Yes	12 (27.3%)	0.000
	No	32 (72.7%)	
Psoriatic arthritis	Yes	2 (4.5%)	0.000
	No	42 (95.4%)	
Family history	Yes	13 (29.5%)	0.000
	No	31 (70.5%)	
Smoking	Yes	18 (40.9%)	0.083
	No	26 (59.1%)	

* Z test for two independent variables.

**Figure 1:**
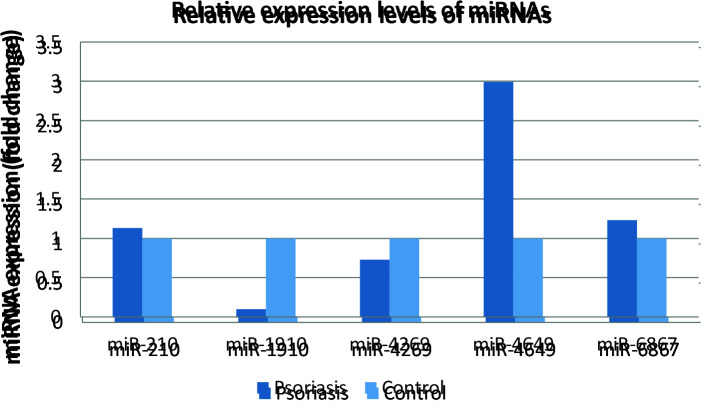
Comparison of miRNA fold change levels in patients with psoriasis and control group.

The mean plasma miR-1910-3p level was significantly lower in the patient group (14.54 ± 2.39) compared to the control group (17.80 ± 0.87) (P = 0.000, fc: 0.10). The mean plasma miR-4649-3p level was significantly higher in the patient group (16.21 ± 1.97) than the control group (14.63 ± 1.82) (P = 0.000, fc: 2.99). The mean plasma miR-4296 level was lower in the patient group (8.72 ± 2.25) compared to the control group (9.17 ± 1.30); however, this difference was not statistically significant (P = 0.255, fc: 0.73). There was no statistically significant difference in the mean plasma levels of miR-210 and miR-6867-5p between the patient and control groups. The mean serum IL-17A levels were significantly higher in the patient group (167.03 ± 374.23 ng/L) than in the control group (42.28 ± 25.51 ng/L) (P = 0.033). A comparison of the plasma miRNAs and IL-17A levels and miRNA fold change levels between the patient and control groups are given in Table 2 and Figure 1, respectively. IL-17A levels significantly differs with the age at disease onset. Serum IL-17A levels were significantly higher in patients with early age at onset of disease (223.78 ± 435.21 ng/L) compared to those with late age at disease onset (31.69 ± 12.14 ng/L) (P = 0.020). 

In logistic regression analyses, it was found that males had an approximately 20-fold higher likelihood to develop the disease (OR = 20.742, P = 0.092). A decrease in plasma miR-1910-3p level was found to increase the likelihood of having the disease by approximately 50-fold (OR = 50.801, P = 0.009) (Table 4).

**Table 4:** Examination of age, sex, miRNA and IL-17A levels by logistic regression analysis.

Clinical and laboratory properties	OR	95% Confidence interval		P*
		Lower value	Upper value	
Age	1.000	0.917	1.090	0.998
Sex	20.742	0.607	709.057	0.092
miR-1910-3p ΔCT	50.801	2.701	955.604	0.009
miR-4649-3p ΔCT	0.355	0.097	1.290	0.116
miR-4296 ΔCT	0.416	0.098	1.759	0.233
miR-210 ΔCT	2.543	0.088	73.395	0.586
miR-6867-5p ΔCT	0.108	0.009	1.264	0.076
IL-17A	1.009	0.999	1.019	0.095

* Logistic regression analysis.

In correlation analysis, a statistically significant negative correlation was found between serum IL-17A and miR-6867-5p expression levels (r: –0.314, P = 0.015). There was also a statistically significant correlation between age at disease onset and the expression level of plasma miR-4649-3p (r: 0.327, P = 0.03). Moreover, a statistically significant negative correlation was shown between IL-17A levels and PASI (r: 0.373, P = 0,013) and age at disease onset (r: –0.382, P = 0.010) (Table 5).

**Table 5:** Correlations between plasma miRNA levels, IL-17A, disease onset age, disease duration, disease severity (PASI).

	Disease onset age	PASI	IL17-A
miR-1910-3p	.100	.032	0.101
miR-4649-3p	.327*	.127	0.092
miR-4296	–.214	–.033	0.076
miR-6867-5p	–.208	.064	–.314*
IL-17A	–.382**	.373**	1

**Figure 2:**
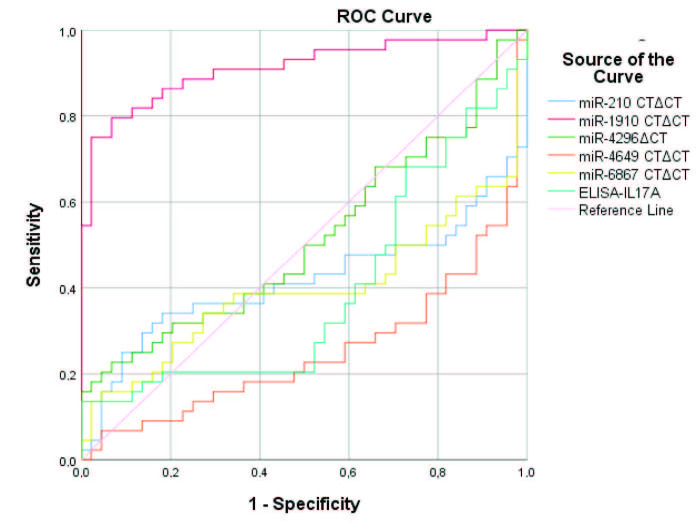
Evaluation of plasma miRNA and serum IL-17A levels by ROC curve analysis.

In Receiver Operating Characteristic Curve (ROC) analysis, the plasma miR-1910-3p level was found to be significantly better at discriminating psoriasis patients from healthy controls (AUC = 0.912 (0.848 - 0.975), p = 0.000) (Table 6, Figure 2). The cut-off value of plasma miR-1910-3p level was 17.1114 (Sensitivity = 0.864, Specificity = 0.818). The sensitivity of a test is its ability to identify the patient as a patient; whereas specificity can be defined as the power to identify healthy people as healthy in general. The cut-off value of the other parameters studied was not determined, since the predictive power of the disease was below 50%.

**Table 6:** Evaluation of plasma miRNA and serum IL-17A levels by ROC curve analysis.

	AUC*	P*
miR-1910-3p ΔCT	0.912	0.000
miR-4649-3p ΔCT	0.255	0.000
miR-4296 ΔCT	0.515	0.809
miR-210 ΔCT	0.416	0.176
miR-6867-5p ΔCT	0.398	0.098
IL-17A	0.394	0.087

* Area under the curve (AUC).

## 5. Discussion

IL-17A, a member of the IL-17 family of cytokines, plays an important role in the pathogenesis of psoriasis by increasing the production of proinflammatory cytokines [6]. A significant increase in serum and tissue levels of IL-17A in patients with psoriasis has been reported [3,6,7]. In the current study, the serum IL-17A level (167.03 ± 374.23 ng/L) was significantly higher in psoriatic patients compared to the healthy controls (42.28 ± 25.51 ng/L) (P = 0.033). Although it was reported that IL-17 levels increase with increasing disease severity [8–11], there are not enough studies examining the relationship between serum IL-17A levels and the severity of psoriasis. In a recent study, it has been reported that IL-17A levels in the skin and serum increase as PASI increases; moreover, serum IL-17A levels were observed to be significantly higher in the patient group with active psoriasis which have new lesion development in the last month compared to patients with stable disease [7]. In the current study, the severity of psoriasis was significantly correlated with the increase in serum IL-17A levels (r: 0.373, P = 0.013). Therefore, it can be suggested that serum IL-17A levels increase as the severity of the disease increases, alternately, enhanced serum IL-17A levels may increase inflammation and disease severity. Our results suggest that serum IL-17A level can be used as an objective parameter to determine the severity of psoriasis.

In our study, serum IL-17A levels were significantly higher in patients with early onset psoriasis (223.78 ± 435.21 ng/mL) than those with late onset group (31.69 ± 12.14 ng/mL), respectively (P = 0.020). In addition, serum IL-17A levels were observed to be increased significantly as the age at disease onset decreased (r: –0.382 P = 0.005). To the best of our knowledge, there has been no study that show a correlation between serum IL-17A levels and age at disease onset. As the disease progresses more severely in the patients with early-onset disease, it is likely that the mean serum IL-17A levels are likely higher in these patients. 

miRs usually bind to the 3’UTR region on the target mRNA, causing either the degradation of the mRNA sequence or an inhibition of its translation [5]. Previous studies have evaluated the levels of miR-1266, miR-340 and miR-197, which are thought to act via IL-17A in patients with psoriasis [12–15]. In two studies, miR1266 were found to be significantly higher in plaque psoriasis patients than in healthy controls and showed a correlation with PASI, suggested that the effect of miR-1266 on the pathogenesis of psoriasis was not through a regulation of IL-17A expression but through a more upstream signaling mechanism or miR-1266 is not likely to regulate IL-17A expression directly but may be involved in the pathogenesis of psoriasis by regulating other target molecules [12–13]. Bian et al. have shown that miR-340 can bind specifically to the 3’UTR region of IL-17A and reduce endogenous IL-17A expression in mouse skin with psoriasis [14]. Elharrar et al. reported that miR-197 expression was lower in psoriatic skin keratinocytes than in the healthy skin, suggesting that a decrease in the suppression of the IL-17A pathway may lead to an accelerated proinflammatory process [15]. In our study, plasma levels of miR-1910-3p (targets IL-17A mRNA) were found to be significantly lower in the patient group compared to the controls (P = 0.000, fc: 0.10). Additionally, we found that a decrease in plasma miR-1910-3p levels could predict nearly a 50-fold increase in the risk of developing the disease (OR = 50.801, P = 0.009). It may be suggested that decreased levels of miR-1910-3p may lead to the loss of suppression of the IL-17A mRNA and a concomitant increase in the expression of IL-17A protein. This may then accelerate the production of proinflammatory molecules involved in the pathogenesis of psoriasis and lead to enhanced keratinocyte proliferation.

Currently, there are no laboratory-based methods for the diagnosis of psoriasis. A ROC curve analysis is used to determine the power of a particular parameter to distinguish patients from healthy individuals. Our ROC curve analysis indicated that plasma miR-1910-3p levels could be used to distinguish psoriasis patients from healthy controls [AUC = 0.912 (0.848–0.975), P = 0.000]. Furthermore, the ROC curve analysis revealed that the plasma miR-1910-3p levels showed a stronger discriminatory power to distinguish psoriasis patients from controls when compared to previously studied miRs [16–18]. We strongly think that plasma miR-1910-3p levels can be used as a diagnostic biomarker for psoriasis.

Impaired Treg cell function has been suggested as one of the causes of T cell activation in psoriasis. Although the mechanisms for the decline in suppressive functions of Treg cells are still unclear, miRs are thought to play a role [4,19]. miR-4649-3p, is thought to be effective on Treg cells. Although miR-4649-3p has been implicated in different diseases, it has not been studied in the context of psoriasis [20,21]. miR-4649-3p was suggested to inhibit cell proliferation in nasopharyngeal carcinoma by targeting protein tyrosine phosphatase SHP-1 [20]. Additionally, miR-4649-3p was suggested to be a biomarker that can predict the transition to chronic disease in patients with brucellosis [21]. In the current study, the plasma miR-4649-3p level was significantly higher in psoriatic patients compared to the control group (P = 0.000, fc: 2.99). Increased expression of miR-4649-3p in psoriasis may lead to a decrease in Treg cell production (by suppressing FOXP3 gene) causing an exacerbation of immune response. We also found that miR-4649-3p plasma levels decreased significantly as the age at disease onset decreased (r: 0.323, P = 0.03), suggesting that Treg cell development and function are likely to also be regulated by proteins and miRs other than miR-4649-3p in the early onset psoriasis group.

To the best of our knowledge, there are no studies examining the role of miR-4296 in different disease states including psoriasis. In the current study, plasma levels of miR-4296 were lower in the patient group compared to controls, but the difference did not reach statistical significance (P = 0.255, fc: 0.73). In fact, that miR-4296 was expected to show high expression in the patient group as the miRNA was predicted to target the mRNA of FOXP3 with a high target score MicroRNA target prediction database (miRDB). This suggests that the role of miR-4296 in the pathogenesis of psoriasis may be via other relevant target genes rather than directly through the inhibition of FOXP3. In addition, the fact that the patient cohort in the current study had predominantly mild disease and belonged to a different population from previous studies could also have led to distinct outcomes.

Studies have evaluated the expression of miR-210 in psoriatic patients and higher miR210 levels were detected [4,22]. In these studies, it was reported that miR-210 regulated Treg cell functions by targeting FOXP3 in psoriasis and increased levels of miR-210 in psoriasis could skew T cells differentiation to Th17 and Th1, but not to Th2 [4,22]. In contrast, Abdul-Maksoud et al. found significantly lower miR-210 levels in another inflammatory disease RA, and negatively correlated miR-210 levels with clinical and laboratory inflammatory markers [23]. In our study, there was no significant difference in the plasma miR-210 levels between the patient and control groups (P = 0.555, fc: 1.13) suggesting that the association of a single miRNA with the risk of developing a disease can vary significantly between different populations. 

In the present study, no significant difference was found in the plasma levels of miR-6867-5p between the patient and control groups (P = 0.354, fc: 1.23). To the best of our knowledge, miR-6867-5p expression has not been evaluated in patients with psoriasis but has been studied in the context of endometriosis, suggesting regulatory effect of miR-6867-5p on cell proliferation [24]. In our study, there was a significant negative association between the plasma level of miR-6867-5p and serum level of IL-17A (r: -0.315 p = 0.038). The lack of a significant difference in the plasma levels of miR-6867-5p between the patient and control groups together with the presence of a significant negative association between miR-6867-5p and IL-17A in the patient group suggests that miR-6867-5p may directly regulate the IL-17A mRNA rather than the activity of Treg cells in patients with psoriasis.

The severity of the disease was not homogeneously distributed in the patient cohort as the number of patients with mild psoriasis was higher than the number of patients with moderate-severe psoriasis is the limitation of the study. On the other hand, evaluation of circulating levels of miR-1910-3p (predicted to target the IL-17A mRNA) and miR-4649-3p, miR-4296, miR-6867-5p (predicted to target the FOXP3 mRNA that regulates the functions of Treg cells), which had not been studied in patients with psoriasis before, are the strengths of the current study.

Our results show that decreased plasma levels of miR-1910-3p and increased levels of miR-4649-3p may be associated with psoriasis. Particularly, decreased miR-1910-3p levels increase the risk of developing psoriasis by approximately 50-fold, and plasma levels of miR-1910-3p were able to significantly differentiate psoriatic patients from healthy controls. Therefore, plasma levels of miR-1910-3p could be used as a biomarker for the diagnosis of psoriasis. Also miR-1910-3p and miR-4649-3p, could be useful targets for the development of drugs used in the treatment of psoriasis. Further and comprehensive studies that show relevant tissue expressions of miRs in addition to the plasma levels are needed in order to better determine the roles of miRs on cytokines and signaling pathways in the pathogenesis of psoriasis and to use them as target molecules for therapeutic purposes.

## Informed consent

Written informed consents were obtained from all participents.
